# The Fall of Icarus: Post-psychotic depression - Apropros a clinical case

**DOI:** 10.1192/j.eurpsy.2023.1787

**Published:** 2023-07-19

**Authors:** S. Jesus, A. R. Costa, G. Simões, M. Almeida, A. Tarelho, P. Garrido

**Affiliations:** Departamento de Psiquiatria e Saúde Mental, Centro Hospitalar do Baixo Vouga, Aveiro, Portugal

## Abstract

**Introduction:**

Depressive symptoms occur in different phases of psychosis, including prodromal, acute and post-psychotic. Post-psychotic depression (PPD) is a phenomenon that presents as a diagnostic and therapeutic challenge. Having been ascribed various descriptions in the past, PPD has been used in a broad manner to describe depressive symptoms that appear in patients with history of psychosis. PPD unveils itself as a separate nosological entity, differing from the adverse effects typically associated with antipsychotics, the negative symptoms of psychosis, and other psychiatric disorders that present with both psychotic and depressive symptoms (e.g. bipolar disorder, schizoaffective disorder, or psychotic depression).

**Objectives:**

The authors present a case of a 64 year-old man hospitalized due to inaugural psychosis with persecutory and grandiose delusions as well as auditory hallucinatory activity, who began to develop a depressive clinical picture whilst under treatment. A brief discussion on post-psychotic depression, from its clinical presentation to its treatment and implications in prognosis is also presented.

**Methods:**

A brief non-systematized literature review using the *Pubmed* platform as well as presentation of a clinical case.

**Results:**

Depressive complaints are a common complication of psychotic episodes, with the literature estimating that approximately a quarter of psychotic patients present with PPD. Although typically described in association with schizophrenia, recent literature describes PPD occurring alongside other psychotic presentations, including first-episode psychosis. A division between affect and psychosis has been attempted in terms of psychiatric classification, however, the blurred lines between the two continue to contribute to difficulties in differential diagnosis. This becomes a challenge when distinguishing between extrapyramidal symptoms associated with antipsychotics, negative symptoms (i.e apathy, abulia and alogia) and psychiatric disorders with affective-psychotic overlap. Having only recently been considered a distinct clinical entity in psychiatric classification systems, research on its etiology, course, treatment and prognosis are scarce. In regards to the previously described patient, a depressive disorder whilst in treatment for psychosis was identified, and through early recognition of the symptoms treatment with an antidepressant was initiated with favourable response.

**Conclusions:**

PPD is a relatively common phenomenon which is gaining more attention in recent literature. As classifications have begun to consider PPD as a distinct clinical entity, as well as unifying defining criteria, further studies can be developed so as to clarify aspects which remain to be defined. The clinician should be aware of this entity as well as the potentially confounding symptom presentations, so as to provide adequate early treatment thus contributing to improved patient outcomes.

**Disclosure of Interest:**

None Declared

